# Factors associated with the consumption of medicinal plants for the prevention of COVID-19 in peruvian population: a cross-sectional study

**DOI:** 10.17843/rpmesp.2024.411.13265

**Published:** 2024-03-25

**Authors:** Fabricio Ccami-Bernal, Cristhian Rojas-Miliano, David R. Soriano-Moreno, Daniel Fernández-Guzmán, Carlos Quispe-Vicuña, Enrique A. Hernández-Bustamante, Elvira G. Zamora-Huaringa, Wendy Nieto-Gutiérrez

**Affiliations:** 1 Universidad Nacional de San Agustín de Arequipa, Arequipa, Peru. Universidad Nacional de San Agustín Universidad Nacional de San Agustín de Arequipa Arequipa Peru; 2 Universidad Nacional del Centro del Perú (UNCP), Huancayo, Peru. Universidad Nacional del Centro del Perú Universidad Nacional del Centro del Perú (UNCP) Huancayo Peru; 3 Unidad de Investigación Clínica y Epidemiológica, Escuela de Medicina, Universidad Peruana Unión, Lima, Peru. Universidad Peruana Los Andes Unidad de Investigación Clínica y Epidemiológica Escuela de Medicina Universidad Peruana Unión Lima Peru; 4 Carrera de Medicina Humana, Universidad Científica del Sur, Lima, Peru Universidad Científica del Sur Carrera de Medicina Humana Universidad Científica del Sur Lima Peru; 5 Sociedad Científica San Fernando, Universidad Nacional Mayor de San Marcos, Lima, Peru. Universidad Nacional Mayor de San Marcos Sociedad Científica San Fernando Universidad Nacional Mayor de San Marcos Lima Peru; 6 Sociedad Científica de Estudiantes de Medicina de la Universidad Nacional de Trujillo, Trujillo, Peru. Universidad Nacional de Trujillo Sociedad Científica de Estudiantes de Medicina Universidad Nacional de Trujillo Trujillo Peru; 7 Facultad de Medicina Humana, Universidad Ricardo Palma, Lima, Peru. Universidad Ricardo Palma Facultad de Medicina Humana Universidad Ricardo Palma Lima Peru; 8 Unidad de Investigación para la Generación y Síntesis de Evidencias en Salud, Universidad San Ignacio de Loyola, Lima, Peru. Universidad San Ignacio de Loyola Unidad de Investigación para la Generación y Síntesis de Evidencias en Salud Universidad San Ignacio de Loyola Lima Peru

**Keywords:** COVID-19, SARS-CoV-2, Medicinal plants, Herbal medicine, Traditional medicine

## Abstract

**Objectives.:**

Determine the factors associated with the consumption of medicinal plants as a preventive measure against COVID-19 in the Peruvian population.

**Materials and methods.:**

A population over 18 years of age, living in Peru and without a history of COVID-19 disease, was evaluated. The factors associated with the consumption of medicinal plants were evaluated using a Poisson regression model with robust variances.

**Results.:**

Of the 3231 participants included, 84.6% were young adults (18-29 years old), 62.7% were women, and 59.7% consumed a medicinal plant to prevent COVID-19 infection. The factors associated with the consumption of medicinal plants to prevent COVID-19 infection were residing in the Peruvian highlands, having had a family member diagnosed with COVID-19, having had a family member die from COVID-19, considering their family to be at increased risk of infection, having used medications or chlorine dioxide to prevent COVID-19, having medical information as the main source of information about COVID-19, thinking that medicinal plants are effective in preventing COVID-19 disease, or not being informed about their effectiveness.

**Conclusion.:**

Sixty percent of the participants reported having consumed a medicinal plant to prevent COVID-19. Authorities must apply communication strategies about the implications of consuming medicinal plants, prioritizing population groups with higher consumption patterns.

## INTRODUCTION

The COVID-19 pandemic has wreaked havoc on all regions of the world ^1^^,^^2^, fostering an atmosphere of fear and uncertainty among the population ^3^. This has prompted people to seek various disease prevention measures ^4^. For instance, using medicines, medicinal plants, and other remedies is a common practice to guard against SARS-CoV-2 infection, with the prevalence of such practices varying across populations due to social and cultural factors ^5^.

Before the COVID-19 pandemic, in the health systems of low- and middle-income countries, with limited resources and scarce health personnel, there was a population belief in trusting and practicing alternative medicines such as the consumption of medicinal plants ^6^^).^ This is the case of Peru, which is characterized by a great wealth and diversity of medicinal plants and a culture where their use predisposition and acceptance are widespread in the population and even in the health system ^7^.

Traditionally, medicinal plants have been used to prevent and treat respiratory infections ^8^. Some studies propose that certain medicinal plants may have antiviral properties ^9^. However, there is no conclusive evidence that they can be used to treat the symptoms of COVID-19 ^10^. Indiscriminate use of medicinal plants could be harmful to one’s health ^11^^,^^12^. Therefore, this study was developed to determine the factors associated with the consumption of medicinal plants for the prevention of COVID-19 in the Peruvian population.

**KEY MESSAGES**

Motivation for the study: No study conclusively recommends the use of medicinal plants to treat COVID-19 symptoms, and their indiscriminate use may present health risks.

Main findings: Sixty percent of participants consumed medicinal plants to prevent COVID-19. This was particularly true for those living in the Peruvian highlands and individuals with family members diagnosed or deceased from COVID-19, who perceive a higher risk of infection and use medications or chlorine dioxide as preventive measures.

Implications: Communication strategies emphasizing validated preventive practices and educating about the risks of consuming medicinal plants should be tailored to the predominant characteristics of the consumer.

## MATERIALS AND METHODS

### Study design and population

A secondary analysis was conducted on a database from a previously reported study ^13^ that aimed to describe preventive and control practices for SARS-CoV-2 transmission in the Peruvian population. The corresponding author can provide access to the database upon request. Participants were recruited through non-probabilistic snowball sampling. The study evaluated the Peruvian population over 18 years of age residing in Peru. Data collection occurred in September 2020 through a virtual survey distributed via social networks like Facebook, WhatsApp, Instagram, and Telegram. The analysis included participants with no prior history of COVID-19 and who responded to the question of interest.

### Instrument

The authors created a survey to gather data for the main study. The formulation of the questionnaire was grounded on previous study reports and underwent evaluation and approval by experts. Additionally, it underwent a pilot test to assess its validity, yielding a Cronbach's alpha of 0.70 (14). The instrument was divided into three sections: the first section collected general, sociodemographic, and epidemiological data; the second section focused on prevention practices and perspectives for individuals without a previous diagnosis of COVID-19; and the third section addressed prevention practices and perspectives for individuals with a prior diagnosis of COVID-19. This study solely examined variables from the first two sections. The survey can be accessed in supplementary material 1.

### Variable of interest

The primary outcome of interest was self-reported consumption of medicinal plants for the prevention of COVID-19. This was assessed using a multiple-choice question: “What medicinal plants do you use exclusively to prevent COVID-19?” Response options included the common names of commonly consumed medicinal plants in Peru, such as eucalyptus (*Eucalyptus globulus*), mallow (*Malva sylvestris*), plantain (*Plantago major*), quinine (*Cinchona officinalis*), lemon verbena (*Aloysia citrodora*), cypress (*Cupressus macrocarpa*), soursop leaves (*Annona muricata*), lemon leaves (*Citrus limon*), ginger (*Zingiber officinale*), matico (*Piper aduncum*), and garlic (*Allium sativum*). If a respondent reported consuming at least one of the listed plants, it was considered indicative of use for preventive purposes.

### Other variables

The study evaluated various sociodemographic and health-related factors: Demographics: Sex (female, male), age group (18-29 years, 30 years or older), marital status (single, married/cohabitating), educational level (secondary or lower, higher), region of residence (coast, mountains, jungle), reported area of residence (urban, rural), employment status (employed, unemployed), and social class (low, upper-middle). Health factors: Presence of comorbidities (no, yes), family member with comorbidity (no, yes), family member diagnosed with COVID-19 (no, yes), family member who died from COVID-19 (no, yes). Information and Practices: Source of information about COVID-19 (social networks, press media, medical information, friends/family), opinion on the use of medicinal plants for COVID-19 prevention (not effective, effective, not reported), use of medication for COVID-19 prevention (no, yes), use of chlorine dioxide for COVID-19 prevention (no, yes). Perspectives: Perspectives on COVID-19 (not included in the current analysis).

### Statistical analysis

Absolute and relative frequencies were calculated, as well as the chi-square test for comparison between groups. For the analysis of the associated factors, the Poisson regression model with robust variances was used and the prevalence ratios (PR) with their respective 95% confidence intervals (95% CI) were calculated. Values of p<0.05 were considered statistically significant. Collinearity was assessed using the variance inflation factor, with a threshold set at a value greater than 10 indicating the presence of collinearity. The adjusted model was built incorporating all variables since our study adopted an epidemiological approach. This decision was based on consideration of variables that were anticipated to be associated with the outcome, supported by previous literature that had explored this association ^14^^-^^16^.

Because the study examined associated factors, the evaluation of sample power was based on the main independent variable, which the authors identified as residence type. Anticipating a difference between categories of 38.6% (rural: 69.3% vs. urban: 30.7%) and with a confidence level of 95%, we found that the statistical power exceeded 80%. Data analysis was performed with Stata v.16.0 software.

### Ethical aspects

The primary study protocol was evaluated and approved by the institutional ethics committee of the Universidad Peruana Unión (Code: 2020-CEUPeU-00020). In addition, it was registered on the Health Research Projects platform (PRISA) in Peru with the code EI00000001472. The survey was anonymous and consent was obtained from respondents for the use of the data in future research. The same authors participated in the initial research and had authorization to access the database.

## RESULTS

A total of 3630 participants were involved in the primary study. After excluding 399 participants who reported having COVID-19, a final sample of 3231 participants was included for analysis. Among the participants in the final sample, 84.6% were young, 62.7% were women, 47.7% lived in the mountains, 84.8% lived in an urban area, and 59.0% had a higher education level (Table 1).

**Table 1:** Characteristics of the population studied according to consumption of medicinal plants as prevention for COVID-19, Peru, 2020 (N=3231)

Variables		Total N (%)	Consumption of medicinal plants		
			No	Yes	p*
			n (%)	n (%)	
Sex					
	Male	1204 (37.3)	545 (41.8)	659 (34.2)	<0.001
	Female	2027 (62.7)	758 (58.2)	1269 (65.8)	
Age (years)					
	Young (18 to 29 years old)	2733 (84.6)	1129 (86.7)	1604 (83.2)	0.008
	Adult and older adults (≥30 years old)	498 (15.4)	174 (13.3)	324 (16.8)	
Marital status					
	Single	2832 (87.6)	1158 (88.9)	1674 (86.8)	0.083
	Married or partner	399 (12.4)	145 (11.1)	254 (13.2)	
Education degree					
	High school or lower	773 (23.9)	357 (27.4)	416 (21.6)	<0.001
	Higher education	2458 (76.1)	946 (72.6)	1512 (78.4)	
Region					
	Coast	1581 (48.9)	733 (56.3)	848 (44.0)	<0.001
	Highland	1369 (42.4)	450 (34.5)	919 (47.7)	
	Jungle	281 (8.7)	120 (9.2)	161 (8.3)	
Residence					
	Urban	2741 (84.8)	1155 (88.6)	1586 (82.3)	<0.001
	Rural	490 (15.2)	148 (11.4)	342 (17.7)	
Work status					
	Unemployed	2509 (77.6)	1015 (77.9)	1494 (77.5)	0.785
	Employed	722 (22.4)	288 (22.1)	434 (22.5)	
Social status					
	Low	1326 (41.0)	632 (48.5)	694 (36.0)	<0.001
	Middle-High	1905 (59.0)	671 (51.5)	1234 (64.0)	
Healthcare professional in family					
	No	1257 (38.9)	412 (31.6)	845 (43.8)	<0.001
	Healthcare science student	518 (16.0)	199 (15.3)	319 (16.6)	
	Yes	1456 (45.1)	692 (53.1)	764 (39.6)	
Source of information about COVID-19					
	Social networks	1098 (34.0)	363 (27.9)	735 (38.1)	<0.001
	Press media	1173 (36.3)	412 (31.6)	761 (39.5)	
	Medical information	847 (26.2)	490 (37.6)	357 (18.5)	
	Friends and family members	113 (3.5)	38 (2.9)	75 (3.9)	
Comorbidities for COVID-19					
	No	2757 (85.3)	1114 (85.5)	1643 (85.2)	0.827
	Yes	474 (14.7)	189 (14.5)	285 (14.8)	
Family members with comorbidities for COVID-19					
	No	1587 (49.1)	638 (49.0)	949 (49.2)	0.886
	Yes	1644 (50.9)	665 (51.0)	979 (50.8)	
Family members diagnosed with COVID-19					
	No	2397 (74.2)	1060 (81.4)	1337 (69.3)	<0.001
	Yes	834 (25.8)	243 (18.6)	591 (30.7)	
Familiar died by COVID-19					
	No	3188 (98.7)	1295 (99.4)	1893 (98.2)	0.003
	Yes	43 (1.3)	8 (0.6)	35 (1.8)	
Do you consider COVID-19 a dangerous and deadly disease?					
	Nothing or almost nothing	750 (23.2)	338 (25.9)	412 (21.4)	0.003
	A lot	2481 (76.8)	965 (74.1)	1516 (78.6)	
Do you consider that using medications, plants or other substances protects you from COVID-19?					
	Nothing or almost nothing	2610 (80.8)	1219 (93.6)	1391 (72.1)	<0.001
	A lot	621 (19.2)	84 (6.4)	537 (27.9)	
Do you consider that you have a higher risk of getting COVID-19?					
	Nothing or almost nothing	2419 (74.9)	1011 (77.6)	1408 (73.0)	0.003
	A lot	812 (25.1)	292 (22.4)	520 (27.0)	
Do you consider that your family is exposed to a higher risk of getting COVID-19?					
	Nothing or almost nothing	1764 (54.6)	750 (57.6)	1014 (52.6)	0.005
	A lot	1467 (45.4)	553 (42.4)	914 (47.4)	
Do you consider that there are many cases of COVID-19 in your community?					
	Nothing or almost nothing	1788 (55.3)	703 (53.9)	1085 (56.3)	0.192
	A lot	1443 (44.7)	600 (46.1)	843 (43.7)	
Do you consider that you carry out prevention measures against COVID-19 properly?					
	Nothing or almost nothing	988(30.6)	373 (28.6)	615 (31.9)	0.048
	A lot	2243 (69.4)	930 (71.4)	1313 (68.1)	
Opinion on the use of medicinal plants to prevent COVID-19					
	It’s effective	1611 (49.9)	256 (19.7)	1355 (70.3)	<0.001
	It’s not effective	822 (25.4)	480 (36.8)	342 (17.7)	
	Not informed	798 (24.7)	567 (43.5)	231 (12.0)	
Use of medications to prevent COVID-19					
	No	2517 (77.9)	1208 (92.7)	1309 (67.9)	<0.001
	Yes	714 (22.1)	95 (7.3)	619 (32.1)	
Use of chlorine dioxide to prevent COVID-19					
	No	2972 (92.0)	1253 (96.2)	1719 (89.2)	<0.001
	Yes	259 (8.0)	50 (3.8)	209 (10.8)	

*Calculated with chi2 test; significant p-value in bold (p<0.05).

Regarding the perception of COVID-19, 76.8% of those surveyed considered that it was very dangerous and deadly, 74.9% thought that they had low or no risk of getting sick from COVID-19, 45.4% considered that their relatives were exposed to a greater risk of becoming infected, 44.7% stated that there were many cases of COVID-19 in their community and 49.9% considered that the consumption of medicinal plants was effective in preventing transmission of COVID-19 (Table 1).

It was reported that 59.7% consumed at least one medicinal plant to prevent COVID-19, the most consumed was eucalyptus (77.7%), *kion* (66.7%) and *matico* (44.6%), The description of the total medicinal plants consumed is presented in supplementary material 2. The highest proportion of consumers were women (65.8%, p<0.001), young people (18-29 years) (83.2%, p= 0.008), with higher education (78.4%, p<0.001), from the mountain region (47.7%, p<0.001), with urban residence (82.3%, p<0.001), middle class- high (64.0%, p<0.001), who did not have a health professional within the family unit (43.8%, p<0.001), acquired information about COVID-19 through social networks (38.1%, p<0.001), did not have a family member diagnosed (69.3%, p<0.001) or died of COVID-19 (98.2%, p=0.003). In addition, those who strongly considered COVID-19 as a dangerous and deadly disease (78.6%, p=0.003), who did not consider at all or little that taking medications, plants or other substances protect against getting sick from COVID-19 (72. 1%, p<0.001), those who considered nothing or little that they were at greater risk of contracting COVID-19 (73.0%, p=0.003), those who considered nothing or little that their family was exposed to a greater risk of contracting COVID-19 (52.6%, p=0.005), who considered that they adequately carried out preventive measures against COVID-19 (68.1%, p=0.048), who considered that the consumption of medicinal plants to prevent COVID -19 was effective (70.3%, p<0.001) and that they did not use medications (67.9%, p<0.001) or chlorine dioxide to prevent COVID-19 (89.2%, p<0.001) ( Table 1).

Regarding the associated factors in the adjusted analysis, we found that the consumption of medicinal plants was associated with residing in the Peruvian mountains (PRa 1.13, 95% CI: 1.07 - 1.19), having had a close relative diagnosed with COVID-19 (PRa 1.07, 95% CI: 1.01 - 1.12), having had a family member die from COVID-19 (PRa 1.26, 95% CI: 1.08 - 1.45), consider that their family is at greater risk of infection (PRa 1.06, 95% CI: 1.01 - 1.12), having used medication to prevent COVID-19 (PRa 1.33, 95% CI: 1.27 - 1.39) or having used chlorine dioxide to prevent COVID-19 (PRa 1.13, 95% CI: 1.06 - 1.21), having medical information as a primary source of information about COVID-19 (PRa 0 .81, 95% CI: 0.74 - 0.87), and think that medicinal plants are effective in preventing COVID-19 (PRa 1.02, 95% CI: 0.55 - 0.66) as well as not being informed of its effectiveness (PRa 0.40, 95% CI: 0.36 - 0.45) (Table 2).

**Table 2:** Factores asociados al consumo de plantas medicinales como prevención de COVID-19 en una población peruana, 2020 (N=3231).

Variables		Crude PR	95% CI	Adjusted PR	95% CI
Sexo					
	Femenino	1.00		1.00	
	Masculino	0..87	0.82 - 0.93	0.96	0.91 - 1.01
Age					
	Young (18 to 29 years old)	1.00		1.00	
	Adult and older adults (≥30 years old)	1.11	1.03 - 1.19	1.07	0.98 - 1.17
Estado civil					
	Single	1.00		1.00	
	Married or partner	1.08	0.99 - 1.17	0.96	0.88 - 1.05
Education degree					
	High school or lowerr	1.00		1.00	
	Higher education	1.14	1.06 - 1.23	1.06	0.99 - 1.12
Region					
	Coast	1.00		1.00	
	Highland	1.25	1.18 - 1.33	1.13	1.07 - 1.19
	Jungle	1.07	0.96 - 1.19	0.91	0.83 - 1.00
Residence					
	Urban	1.00		1.00	
	Rural	1.21	1.13 - 1.29	1.00	0.94 - 1.06
Work status					
	Unemployed	1.00		1.00	
	Employed	1.01	0.94 - 1.08	0.93	0.87 - 1.00
Social status					
	Low	1.00		1.00	
	Middle-High	1.24	1.16 - 1.32	1.03	0.98 - 1.09
Healthcare professional in family					
	No	1.00		1.00	
	Healthcare science student	0.92	0.85 - 0.99	0.98	0.92 - 1.05
	Yes	0.78	0.73 - 0.83	0.94	0.89 - 1.00
Source of information about COVID-19					
	Social networks	1.00		1.00	
	Press media	0.97	0.91 - 1.03	0.98	0.93 - 1.03
	Medical information	0.63	0.58 - 0.69	0.81	0.74 - 0.87
	Friends and family members	0.99	0.86 - 1.14	0.94	0.83 - 1.06
Comorbidities for COVID-19					
	No	1.00		1.00	
	Yes	1.01	0.93 - 1.09	0.97	0.90 - 1.04
Family members with comorbidities for COVID-19					
	No	1.00		1.00	
	Yes	1.00	0.94 - 1.05	1.01	0.96 - 1.06
Family members diagnosed with COVID-19					
	No	1.00		1.00	
	Yes	1.27	1.20 - 1.34	1.07	1.01 - 1.12
Familiar died by COVID-19					
	No	1.00		1.00	
	Yes	1.37	1.18 - 1.59	1.26	1.08 - 1.45
Do you consider COVID-19 a dangerous and deadly disease?					
	Nothing or almost nothing	1.00		1.00	
	A lot	1.11	1.04 - 1.20	1.05	0.98 - 1.12
Do you consider that using medications. plants or other substances protects you from COVID-19?					
	Nothing or almost nothing	1.00		1.00	
	A lot	1.62	1.55 - 1.70	1.17	1.11 - 1.22
Do you consider that you have a higher risk of getting COVID-19?					
	Nothing or almost nothing	1.00		1.00	
	A lot	1.10	1.03 - 1.17	1.05	0.99-1.12
Do you consider that your family is exposed to a higher risk of getting COVID-19?					
	Nothing or almost nothing	1.00		1,00	
	A lot	1.08	1.02 - 1.15	1.06	1.01 - 1.12
Do you consider that there are many cases of COVID-19 in your community?					
	Nothing or almost nothing	1.00		1.00	
	A lot	0.96	0.91 - 1.02	0.96	0.91 - 1.01
Do you consider that you carry out prevention measures against COVID-19 properly?					
	Nothing or almost nothing	1.00		1.00	
	A lot	0.94	0.89 - 1.00	0.96	0.91 - 1.01
Opinion on the use of medicinal plants to prevent COVID-19					
	It’s effective	1.00		1.00	
	It’s not effective	0.49	0.45 - 0.54	0.60	0.55 - 0.66
	Not informed	0.34	0.31 - 0.38	0,40	0.36 - 0.45
Use of medications to prevent COVID-19					
	No	1.00		1.00	
	Yes	1.67	1.59 - 1.75	1.33	1.27 - 1.39
Use of Chlorine Dioxide to prevent COVID-19					
	No	1.00		1.00	
	Yes	1.40	1.30 - 1.49	1.13	1.06 - 1.21

PR: Prevalence ratio; 95% CI: 95% confidence interval.

## DISCUSSION

Herbal medicine has been proposed as an alternative in the prevention and control of viral infectious diseases such as SARS-CoV and as an alternative for the management of COVID-19 ^17^. However, the information provided by studies that assess its effectiveness is insufficient or of low quality, and more studies are needed to recommend its use as prevention ^18^. In Peru, a country with great historical, cultural, and natural wealth and diversity, the consumption of medicinal plants has been the main cultural practice linked to health care ^19^.

Our study found that over half of the population reported consuming a medicinal plant to prevent COVID-19. This finding aligns with previous studies conducted in the Peruvian population before the pandemic ^18^^,^^20^. However, the reported consumption rate (50%) is lower than the 80% found in a study from Cusco, Peru ^15^ and higher than the rates of 14.1% and 7.2% reported among Facebook users from Peru and Latin America, respectively ^21^. These variations can be explained by considering the geographical context. The Cusco study was conducted in an Andean region, traditionally characterized by higher consumption of medicinal plants. The discrepancies with the second study might be due to differences in the studied populations and data collection methods.

Similarly, our findings align with those reported in Bangladesh ^14^. However, they differ from those observed in Saudi Arabia ^22^, where 57.6% and 22.1% of participants in the respective studies consumed medicinal plants to prevent COVID-19. This variation suggests that the belief in the preventative properties of medicinal plants might be linked to cultural and social factors. These factors include the level of education, occupation, and economic income of the participants, as evidenced by previous research ^18^^,^^23^. Additionally, greater accessibility and lower cost compared to pharmaceutical products ^24^ likely contribute to increased consumption of medicinal plants by those who believe in their effectiveness.

In our study, we found that living in the mountains was associated with a higher consumption of medicinal plants. This is likely because their use and knowledge for therapeutic purposes is an ancestral practice ^24^, passed down through generations ^25^, forming an important cultural component. Ethnobotanical studies report that knowledge and use of medicinal plants directly correlate with the proximity of people to available sources ^26^^,^^27^. Therefore, considering that a large percentage of the medicinal plants used in Peru are found in the mountains ^28^, it is expected that the population living there would show a greater consumption of plants for medicinal purposes.

On the other hand, our findings revealed that employment was associated with a lower consumption of medicinal plants. This aligns with a Mexican study where unemployment correlated with a higher intake of medicinal plants ^29^. While medicinal plants are generally inexpensive ^30^, access to work and potentially greater financial resources might lead people to favor more expensive, yet heavily advertised, preventative measures. Similarly, illness anxiety could be a mediating factor in this association. Employed individuals, with greater economic stability and access to healthcare resources, might experience less health anxiety ^31^, potentially resulting in lower consumption of medicinal plants.

The media plays a critical role in raising public awareness about COVID-19, but it can also generate panic and spread misinformation, leading people to adopt measures with little or no scientific backing ^32^^,^^33^. Our study found that using medical information as the primary source for COVID-19 knowledge was associated with lower consumption of medicinal plants for prevention. In Peru, while over three-quarters of patients reportedly use medicinal plants, a minimal proportion of doctors recommend them ^7^. This likely stems from medical training primarily focused on Western medicine, resulting in minimal inclusion of medicinal plants in their recommendations. Consequently, we expected that having health professionals in the family unit would also be associated with lower consumption of medicinal plants.

On the other hand, having a family member diagnosed with COVID-19, especially if that family member died, was associated with a greater consumption of medicinal plants. This finding aligns with a similar study conducted in Cusco ^15^. These situations likely heighten the population’s fear and concern about the disease, potentially prompting them to adopt additional preventative measures, such as using medicinal plants, even if the effectiveness of these plants is unknown ^34^, as reported in a previous study ^21^.

We also observed that individuals who consumed medications or chlorine dioxide for COVID-19 prevention reported higher consumption of medicinal plants compared to those who did not. This finding aligns with other studies reporting a frequent overlap between medicinal plant and pharmaceutical drug use ^35^^,^^36^. However, this concurrent consumption practice can be risky due to potential interactions between the plants and medications ^37^. While we haven’t identified clinical trials on this specific topic, an in vitro study suggests an increased risk of liver toxicity from interactions between paracetamol, a commonly used medication during the pandemic, and herbal compounds containing furanocoumarins ^38^.

It is important to consider that the effectiveness, safety, and cost-effectiveness of consuming medicinal plants to prevent COVID-19 have not yet been established by high-quality studies ^15^^,^^39^. Therefore, we recommend promoting educational initiatives, such as workshops and talks, that provide the population with information on validated and scientifically supported preventive measures against COVID-19. These initiatives should also address the potential risks associated with consuming medicinal plants, and be tailored to the specific characteristics of the populations most likely to engage in this practice. Future studies are needed to confirm the findings of this research, and qualitative studies would be valuable to gain a deeper understanding of this phenomenon.

This study has some limitations. First, we used a non-probability sampling method, which restricts our ability to generalize the results to the entire population. Second, data collection relied on a virtual questionnaire distributed through social networks. This approach might have primarily attracted participants with access to and knowledge of these platforms, potentially skewing the results towards a specific socio-economic group. However, despite these limitations, our findings shed light on a previously under-addressed aspect of the COVID-19 pandemic in Peru, a country with a rich history and cultural tradition of using medicinal plants.

In conclusion, over 60% of participants reported consuming a medicinal plant to prevent COVID-19. Factors associated with higher consumption included residing in the mountains, having a family member diagnosed or deceased from COVID-19, believing their family was at greater risk of infection, and having used medications or chlorine dioxide for prevention. Understanding this trend is important, as consuming medicinal plants, like any other unproven substance, carries the risk of adverse effects or a false sense of security.
