# Supplementary material for: Factors associated with the consumption of medicinal plants for the prevention of COVID-19 in peruvian population: a cross-sectional study
Source: Rev Peru Med Exp Salud Publica. 2024 Mar 25;41(1):37–45. doi: 10.17843/rpmesp.2024.411.13265 (PMC11149759; doi:10.17843/rpmesp.2024.411.13265)
Supplement: Supplementary material. — Available in the electronic version of the RPMESP. [file rpmesp-41-01-13265-s001.pdf]

MATERIAL SUPLEMENTARIO

Material suplementario 1. Encuesta

SECCIÓN 1: DATOS GENERALES

1. ¿Qué edad tiene? ___ (en años)

2. ¿Cuál es su género?

- Femenino
- Masculino

3. ¿Cuál es su estado civil?

- Soltero
- Casado o conviviente
- Divorciado o separado
- Viudo

4. ¿Qué grado de instrucción tiene?

- Primaria completa o incompleta
- Secundaria incompleta
- Secundaria completa
- Técnico no superior
- Técnico Superior
- Universitario Superior

5. ¿Cuál es su situación laboral?

- Empleado
- Desempleado

6. ¿Qué tipo de seguro médico tiene?

- Ninguno
- SIS (MINSA)
- Seguro Social (EsSalud)
- Clínica privada
- Seguro de fuerzas armadas o policía
- Otro: ______________

7. ¿Dónde vive? (Departamento, ejemplo: Cusco) ____________________

8. ¿Vive actualmente en una zona rural? (Considerar como zona rural un territorio de pocos habitantes y pocas edificaciones donde la principal actividad económica de la zona es la agricultura, ganadería, fruticultura, horticultura, etc.)

- No
- Sí

9. ¿Recibió su familia algún apoyo del gobierno (bonos económicos del gobierno)?

- No
- Sí

10. ¿Tiene algún familiar dentro de su casa trabajando en un hospital?

- Sí, un profesional de salud familiar.
- Sí, soy profesional de la salud.
- No, nadie en mi familia es profesional de la salud.
- No, pero tengo un familiar que es estudiante de ciencias de la salud.
- No, pero soy estudiante de ciencias de la salud.

11. ¿Qué fuente de información utiliza más para informarse de la COVID-19?

- Redes sociales (Facebook, YouTube, Instagram, Twitter, etc.)
- información de amigos, familiares o vecinos (incluidos mensajes de WhatsApp)
- Información de televisión o radio.
- Medios (periódicos, revistas, etc.)
- Información médica (médicos, revistas científicas, etc.)

12. ¿Tiene alguna de las siguientes condiciones de salud?

- Diabetes mellitus
- Presión arterial alta
- Obesidad (Índice de masa corporal >=30)
- Falla renal crónica
- Asma, fibrosis quística o EPOC
- Inmunosupresión (sistema inmunológico debilitado, incluido el cáncer)
- Condición cardíaca grave (insuficiencia cardíaca o antecedentes de ataque cardíaco)
- Está embarazada
- Ninguno

13. ¿Alguien en su hogar tiene alguna de las condiciones de salud enumeradas en la pregunta anterior o hay alguien mayor de 65 años?

- No
- Sí

14. ¿Algún miembro de su hogar fue diagnosticado con COVID-19? (sin considerarse Ud.)

- No
- Sí

15. ¿Le diagnosticaron COVID-19?

- No
- Sí

SECCIÓN 2: PRÁCTICAS Y PERSPECTIVAS SOBRE COVID-19 (sección solo para quienes no reportaron antecedentes de COVID-19)

22. Con qué frecuencia... (Marque con una " **X** ")

PASANTÍA	**NUNCA**	**RARA VEZ**	**A VECES**	**A MENUDO**	**SIEMPRE**
... ¿usa mascarilla al salir de casa?					
... ¿se lleva las manos sucias a los ojos, la nariz o la boca?					
... ¿se cubre la nariz y la boca al estornudar o toser?					
... ¿usa mascarilla facial al salir de casa?					
... ¿mantiene distancia de otras personas en la calle (al menos 1 m)?					
... ¿va acompañado cuando sale a la calle?					
... ¿se lava las manos con agua y jabón al regresar a casa?					
... ¿desinfecta superficies de objetos y lugares personales?					
... ¿da la mano, choca el puño o el codo con sus amigos?					
... ¿desinfecta su ropa al llegar a casa?					
... ¿desinfecta sus zapatos al llegar a casa?					
... ¿desinfecta sus compras al llegar a casa?					
... ¿desinfecta su dinero al llegar a casa?					

23. En las últimas 2 semanas. Cuantas veces...	**0**	**1**	**2**	**3**	**4**	**5**	**6**	**7**	**8**	**9**	**10**	**11**	**12**	**13**	**≥14**
... ¿ha cambiado o renovado su mascarilla?															
... ¿se ha ido de casa?															
... ¿ha ido a mercados, tiendas, centros comerciales o bancos?															
... ¿ha asistido a actividades religiosas, sociales y/o recreativas?															
... ¿ha subido al transporte público (combis, metros, tren, etc.)?															
... ¿ha usado medicamentos para prevenir el COVID-19?															
… ¿ha utilizado plantas medicinales para prevenir el COVID-19?															
… ¿ha utilizado dióxido de cloro para prevenir el COVID-19?															

24. ¿Qué material utiliza más para su mascarilla?

- ☐ Mascarilla casera de 1 o 2 capas
- ☐ Mascarilla casera de 3 a más capas
- ☐ Mascarilla quirúrgica desechable (comprada en farmacia)
- ☐ Mascarilla N-95
- ☐ Máscara Kn-95
- ☐ Respirador con filtro
- ☐ Otro: _________________

25. ¿Cuánto tiempo se tarda en lavarse las manos?

- ____________ segundos

26. ¿Qué medicamentos toma exclusivamente para prevenir la COVID-19?

- ☐ Ninguno
- ☐ Ivermectina para uso humano.
- ☐ Ivermectina para uso veterinario.
- ☐ Hidroxicloroquina
- ☐ Azitromicina o claritromicina
- ☐ Corticosteroides (dexametasona, prednisona, etc.)
- ☐ Paracetamol
- ☐ AINE (ibuprofeno, naproxeno o diclofenaco)
- ☐ Heparina o warfarina (u otro anticoagulante)
- ☐ Otro: ______

- Hojas de limón
- Kión
- Ajo
- Otro: ____

27. ¿Qué fuente principal de información utilizó para el uso de estos medicamentos?

- No usé medicamentos
- Redes sociales (Facebook, YouTube, Instagram, Twitter, etc.)
- Información de amigos, familiares o vecinos (incluidos mensajes de WhatsApp)
- Información de televisión o radio.
- Medios de prensa (periódicos, revistas, etc.)
- Información médica (médicos, revistas científicas, etc.)

28. ¿Qué plantas medicinales utiliza exclusivamente para prevenir la COVID 19?

- Ninguno
- Eucalipto
- Matico
- Malva
- Plátano
- Quinina
- Ciprés
- Hojas de guanábana

29. ¿Qué fuente principal de información utilizó para el uso de estas plantas?

- No usé plantas
- Redes sociales (Facebook, YouTube, Instagram, Twitter, etc.)
- Información de amigos, familiares o vecinos (incluidos mensajes de WhatsApp)
- Información de televisión o radio.
- Medios (periódicos, revistas, etc.)
- Información médica (médicos, revistas científicas, etc.)

30. Como medida preventiva, ¿qué opinas sobre el uso?

	Es efectivo, hace bien	Es efectivo, hace poco daño	No es efectivo, pero no hace daño	No es efectivo y hace daño	No estoy informado de ese tema
Ivermectina					
Otros medicamentos					
Dioxido de cloro					
Plantas medicinales					
La posible vacuna contra el COVID-19					

31. ¿Hasta qué punto considera...

	Mucho	Pequeño	Nada
... ¿que la COVID-19 ha influido negativamente en su vida?			
... ¿cuántas son las medidas preventivas contra la COVID-19 en tu comunidad?			
... ¿que tiene mayor riesgo de contraer COVID-19?			
... ¿que su familia está expuesta a un mayor riesgo de contraer COVID-19?			
... ¿que la COVID-19 es una enfermedad peligrosa y mortal?			
... ¿que lleva a cabo adecuadamente las medidas de prevención frente a la COVID-19?			
... ¿que usar mascarilla le protege de contagiarse de COVID-19?			

... ¿que hay muchos casos de COVID-19 en su comunidad?			
... ¿que las pruebas rápidas son fiables?			
... ¿que tomar medicamentos, plantas u otras sustancias protegen de enfermar de COVID-19?			

Material suplementario 2: Plantas medicinales consumidas para prevención de la COVID-19 en Perú, 2020

Planta medicinal	N	%
Eucalipto	1498	77,7
Kion	1286	66,7
matico	859	44,6
Hojas de limón	271	14,1
Cedrón	122	6,3
Plátano	102	5,3
Ciprés	77	4,0
Quinina	68	3,5
Malva	53	2,7
Hojas de guanábana	45	2,3
Ajo	23	1,2
Otro	248	12,9
