# Supplementary material for: Factors associated with the consumption of medicinal plants for the prevention of COVID-19 in peruvian population: a cross-sectional study
Source: Rev Peru Med Exp Salud Publica. 2024 Mar 25;41(1):37–45. doi: 10.17843/rpmesp.2024.411.13265 (PMC11149759; doi:10.17843/rpmesp.2024.411.13265)
Supplement: Translation

# Translation: Factores asociados con el consumo de plantas medicinales para la prevención de COVID-19 en población peruana: un estudio transversal

**Authors:** Fabricio Ccami-Bernal, Cristhian Rojas-Miliano, David R. Soriano-Moreno, Daniel Fernández-Guzmán, Carlos Quispe-Vicuña, Enrique A. Hernández-Bustamante, Elvira G. Zamora-Huaringa, Wendy Nieto-Gutiérrez

**Affiliations:** 1 Universidad Nacional de San Agustín de Arequipa, Arequipa, Perú.; 2 Universidad Nacional del Centro del Peru, Huancayo, Perú.; 3 Unidad de Investigación Clínica y Epidemiológica, Escuela de Medicina, Universidad Peruana Unión, Lima, Perú.; 4 Carrera de Medicina Humana, Universidad Científica del Sur, Lima, Perú.; 5 Sociedad Científica San Fernando, Universidad Nacional Mayor de San Marcos, Lima, Perú.; 6 Sociedad Científica de Estudiantes de Medicina de la Universidad Nacional de Trujillo, Trujillo, Perú.; 7 Facultad de Medicina Humana, Universidad Ricardo Palma, Lima, Perú.; 8 Unidad de Investigación para la Generación y Síntesis de Evidencias en Salud, Universidad San Ignacio de Loyola, Lima, Perú.

**Keywords:** COVID-19, SARS-CoV-2, Plantas medicinales, Medicina herbaria, Medicina tradicional

## Abstract

**Objetivos:**

. Determinar los factores asociados al consumo de plantas medicinales como prevención de la COVID-19 en la población peruana.

**Materiales y métodos.:**

Se evaluó población mayor de 18 años, residentes en Perú y sin antecedentes de enfermedad por COVID-19. Los factores asociados al consumo de plantas medicinales se evaluaron mediante un modelo de regresión de Poisson con varianzas robustas.

**Resultados.:**

De 3231 participantes incluidos, el 84,6% eran jóvenes (18-29 años), el 62,7% eran mujeres y el 59,7% consumía alguna planta medicinal para prevenir la COVID-19. Los factores asociados al consumo de plantas medicinales para prevenir el contagio de COVID-19 fueron residir en la sierra peruana, haber tenido un familiar diagnosticado con COVID-19, haber tenido un familiar fallecido por COVID-19, considerar que su familia se encuentra en mayor riesgo de infección, haber usado medicamentos o dióxido de cloro para prevenir la COVID-19, tener información médica como principal fuente de información sobre la COVID-19, pensar que las plantas medicinales son efectivas para prevenir la enfermedad COVID-19 o no estar informado sobre su eficacia.

**Conclusión:**

. El 60% de los participantes reportó haber consumido alguna planta medicinal para prevenir la COVID-19. Es importante que las autoridades apliquen estrategias de comunicación sobre lo que implica el consumo de plantas medicinales, priorizando los grupos poblacionales que tienen mayores patrones de consumo.

## INTRODUCCIÓN

La pandemia de COVID-19 ha causado un gran impacto en todas las regiones del mundo ^1^^,^^2^, generando un ambiente de miedo e incertidumbre entre la población ^3^, y ha motivado a las personas a recurrir a diferentes medidas para prevenir la enfermedad ^4^. Por ejemplo, se ha informado que el uso de medicinas, plantas medicinales y otras sustancias son medidas populares para prevenir la infección por SARS-CoV-2, cuya prevalencia de uso varía según factores sociales y culturales de cada población ^5^.

Antes de la pandemia de COVID-19, en los sistemas de salud, de países de ingresos medios y bajos, con recursos limitados y escaso personal de salud, existía una ideología poblacional de confiar y practicar medicinas alternativas como el consumo de plantas medicinales ^6^. Este es el caso del Perú, que se caracteriza por una gran riqueza y diversidad de plantas medicinales y una cultura donde su uso, predisposición y aceptación están generalizados en la población e incluso en el sistema de salud ^7^.

Tradicionalmente, las plantas medicinales se utilizan a menudo para prevenir y tratar infecciones respiratorias ^8^; se ha postulado que algunas de ellas tienen propiedades antivirales ^9^. Sin embargo, ningún estudio tiene evidencia concluyente para recomendar su uso para tratar los síntomas de la COVID-19 ^10^, y por el contrario su uso indiscriminado puede ser perjudicial para la salud de quienes los consumen ^11^^,^^12^. Por lo tanto, el presente estudio fue desarrollado para determinar factores asociados al consumo de plantas medicinales como prevención de la COVID-19 en la población peruana.

**MENSAJE CLAVE**

Motivación para realizar el estudio: Ningún estudio recomienda de manera concluyente el uso de plantas medicinales para tratar los síntomas de la COVID-19, y su uso indiscriminado puede presentar riesgos para la salud.

Principales hallazgos: El 60% de los participantes utilizó plantas medicinales para la prevención de la COVID-19. Especialmente aquellos de la sierra peruana y personas con familiares diagnosticados o fallecidos por COVID-19, que perciben mayores riesgos de contagio y utilizan medicamentos o dióxido de cloro como medidas preventivas.

Implicancias: Las estrategias de comunicación esenciales para las prácticas preventivas validadas y la educación sobre los riesgos del consumo de plantas medicinales deben adaptarse a las características predominantes del consumidor.

## MATERIALES Y MÉTODOS

### Diseño y población de estudio

Se realizó un análisis secundario de una base de datos de un estudio que tuvo como objetivo describir las prácticas preventivas y de control de la transmisión de la infección por SARS-CoV-2 en la población peruana, y cuya metodología ha sido reportada previamente ^13^. La base de datos puede estar disponible previa solicitud al autor corresponsal. Los participantes fueron seleccionados mediante un muestreo no probabilístico por conveniencia. Se evaluó a población peruana mayor de 18 años residente en Perú y los datos se recolectaron a través de una encuesta virtual que se distribuyó de manera virtual a través de redes sociales incluyendo Facebook, WhatsApp, Instagram y Telegram durante septiembre de 2020. Se incluyó a los participantes sin antecedentes de COVID-19 y que respondieron al desenlace de interés.

### Instrumento

Se utilizó una encuesta creada por los autores para recopilar datos del estudio primario. La formulación del cuestionario se basó en reportes de estudios anteriores y se sometió a una evaluación y aprobación por parte de expertos y se sometió a una prueba piloto para evaluar su validez obteniendo un alfa de Cronbach de 0,70 ^14^. El instrumento se subdividió en tres secciones: la primera recogió información sobre datos generales, sociodemográficos y epidemiológicos; la segunda sobre prácticas y perspectivas de prevención de personas sin diagnóstico previo de COVID-19; y la tercera sobre prácticas y perspectivas de prevención de personas con diagnóstico previo de COVID-19. El presente estudio solo analizó variables en las dos primeras secciones. La encuesta se puede encontrar en el material suplementario 1.

### Variable de interés

El resultado de interés fue el consumo de plantas medicinales para la prevención de la COVID-19, el cual se evaluó con una pregunta de opción múltiple: “¿Qué plantas medicinales utiliza exclusivamente para prevenir la COVID-19?”, teniendo como opciones de respuesta los nombres comunes de las siguientes plantas: eucalipto (*Eucalyptus globulus*)*,* malva (*Malva sylvestris*)*,* plátano (*Plantago major*)*,* quinina (*Cinchona officinalis*)*,* cedrón (*Aloysia citrodora* )*,* ciprés (*Cupressus macrocarpa*)*,* hojas de guanábana (*Annona muricata*)*,* hojas de limón (*Citrus limon*)*,* kion (*Zingiber officinale*)*,* matico (*Piper aduncum*)*,* ajo (*Allium sativum*)*,* entre otras plantas de uso medicinal más consumidas en el Perú. Si el encuestado respondió consumir al menos una de las plantas mencionadas se consideró su uso con fines preventivos.

### Otras variables

Se evaluaron otras variables como sexo (femenino, masculino), edad (joven 18-29 años, adulto o adulto mayor ≥30 años), estado civil (soltero, casado o conviviente), nivel educativo (secundario o inferior, superior), región de residencia (costa, sierra, selva), área de residencia reportada (urbana y rural), situación laboral (no trabaja, trabaja), clase social (baja, media-alta), profesional de la salud o estudiante dentro de la unidad familiar (no, estudiante de ciencias de la salud, profesional de la salud), fuente de información sobre COVID-19 (redes sociales, medios de prensa, información médica, amigos y familiares), presencia de comorbilidades (no, sí), algún familiar con comorbilidad (no, sí), un familiar diagnosticado con COVID-19 (no, sí), familiar fallecido por COVID-19 (no, sí), opinión sobre el uso de plantas medicinales para la prevención de la COVID-19 (no efectivo, efectivo, no informado), uso de medicamento para la prevención de la COVID-19 (no, sí), uso de dióxido de cloro para la prevención de la COVID-19 (no, sí) y perspectivas sobre la COVID-19.

### Análisis estadístico

Se calcularon frecuencias absolutas y relativas, así como la prueba de chi cuadrado para comparación entre grupos. Para el análisis de los factores asociados se utilizó el modelo de regresión de Poisson con varianzas robustas y se calcularon las razones de prevalencia (RP) con sus respectivos intervalos de confianza del 95% (IC 95%). Se consideraron estadísticamente significativos los valores de p<0,05. La colinealidad se evaluó utilizando el factor de inflación de la varianza, con un umbral establecido en un valor superior a 10 que indica la presencia de colinealidad. El modelo ajustado se construyó incorporando todas las variables, ya que nuestro estudio adoptó un enfoque epidemiológico. Esta decisión se basó en la consideración de variables que se anticipaba estarían asociadas con el resultado, respaldadas por literatura previa que había explorado esta asociación ^14^^-^^16^.

Dado que el estudio examinó factores asociados, la evaluación del poder de la muestra se basó en la principal variable independiente, que los autores identificaron como residencia. Anticipando una diferencia entre categorías del 38,6% (rural: 69,3% vs. urbano: 30,7%) y con un nivel de confianza del 95%, encontramos que el poder estadístico superaba el 80%. El análisis de los datos se realizó con el software Stata v.16.0.

### Aspectos éticos

El protocolo del estudio primario fue evaluado y aprobado por el comité de ética institucional de la Universidad Peruana Unión (Código: 2020-CEUPeU-00020). Además, fue registrado en la plataforma de Proyectos de Investigación en Salud (PRISA) en Perú con el código EI00000001472. La encuesta fue anónima y se obtuvo el consentimiento de los encuestados para el uso de los datos en futuras investigaciones. El presente estudio fue realizado por los mismos autores que participaron en la investigación inicial, y poseían la autorización para acceder a la base de datos.

## RESULTADOS

En el estudio primario participaron 3630 encuestados y 399 fueron excluidos porque habían informado que tuvieron COVID-19. Como resultado, se incluyó para el análisis una muestra final de 3231 encuestados. Del total de participantes, el 84,6% eran jóvenes, el 62,7% eran mujeres, el 47,7% habitaba en la sierra, el 84,8% residía en una zona urbana, el 59,0% tenía un nivel socioeconómico medio a alto y el 76,1% tenía un nivel educativo superior (Tabla 1).

**Tabla 1:** Características de la población estudiada por consumo de plantas medicinales como prevención de la COVID-19 en Perú, 2020 (N=3231).

Variables		Total n (%)	Consumo de plantas medicinales		
			No	Sí	Valor p*
			n (%)	n (%)	
Sexo					
	Masculino	1204 (37,3)	545 (41,8)	659 (34,2)	<0,001
	Femenino	2027 (62,7)	758 (58,2)	1269 (65,8)	
Edad					
	Joven (18 a 29 años)	2733 (84,6)	1129 (86,7)	1604 (83,2)	0,008
	Adultos y adultos mayores (≥30 años)	498 (15,4)	174 (13,3)	324 (16,8)	
Estado civil					
	Soltero	2832 (87,6)	1158 (88,9)	1674 (86,8)	0,083
	Casado o conviviente	399 (12,4)	145 (11,1)	254 (13,2)	
Grado de educación					
	Escuela secundaria o inferior	773 (23,9)	357 (27,4)	416 (21,6)	<0,001
	Superior	2458 (76,1)	946 (72,6)	1512 (78,4)	
Región					
	Costa	1581 (48,9)	733 (56,3)	848 (44,0)	<0,001
	Sierra	1369 (42,4)	450 (34,5)	919 (47,7)	
	Selva	281 (8,7)	120 (9,2)	161 (8,3)	
Residencia					
	Urbano	2741 (84,8)	1155 (88,6)	1586 (82,3)	<0,001
	Rural	490 (15,2)	148 (11,4)	342 (17,7)	
Situación laboral					
	Desempleado	2509 (77,6)	1015 (77,9)	1494 (77,5)	0,785
	Empleado	722 (22,4)	288 (22,1)	434 (22,5)	
Estatus social					
	Bajo	1326 (41,0)	632 (48,5)	694 (36,0)	<0,001
	Medio-alto	1905 (59,0)	671 (51,5)	1234 (64,0)	
Profesional sanitario en familia					
	No	1257 (38,9)	412 (31,6)	845 (43,8)	<0,001
	Estudiante de ciencias de la salud	518 (16,0)	199 (15,3)	319 (16,6)	
	Sí	1456 (45,1)	692 (53,1)	764 (39,6)	
Fuente de información sobre COVID-19**					
	Redes sociales	1098 (34,0)	363 (27,9)	735 (38,1)	<0,001
	Medios de prensa	1173 (36,3)	412 (31,6)	761 (39,5)	
	Información médica	847 (26,2)	490 (37,6)	357 (18,5)	
Comorbilidades para COVID-19					
	No	2757 (85,3)	1114 (85,5)	1643 (85,2)	0,827
	Sí	474 (14,7)	189 (14,5)	285 (14,8)	
Familiares con comorbilidades para COVID-19					
	No	1587 (49,1)	638 (49,0)	949 (49,2)	0,886
	Sí	1644 (50,9)	665 (51,0)	979 (50,8)	
Familiares diagnosticados con COVID-19					
	No	2397 (74,2)	1060 (81,4)	1337 (69,3)	<0,001
	Sí	834 (25,8)	243 (18,6)	591 (30,7)	
Familiar fallecido por COVID-19					
	No	3188 (98,7)	1295 (99,4)	1893 (98,2)	0,003
	Sí	43 (1,3)	8 (0,6)	35 (1,8)	
¿Considera la COVID-19 una enfermedad peligrosa y mortal?					
	Nada o casi nada	750 (23,2)	338 (25,9)	412 (21,4)	0,003
	Mucho	2481 (76,8)	965 (74,1)	1516 (78,6)	
¿Considera que utilizar medicamentos, plantas u otras sustancias te protege de la COVID-19?					
	Nada o casi nada	2610 (80,8)	1219 (93,6)	1391 (72,1)	<0,001
	Mucho	621 (19,2)	84 (6,4)	537 (27,9)	
¿Considera que tiene mayor riesgo de contagiarse de COVID-19?					
	Nada o casi nada	2419 (74,9)	1011 (77,6)	1408 (73,0)	0,003
	Mucho	812 (25,1)	292 (22,4)	520 (27,0)	
¿Considera que su familia está expuesta a un mayor riesgo de contraer COVID-19?					
	Nada o casi nada	1764 (54,6)	750 (57,6)	1014 (52,6)	0,005
	Mucho	1467 (45,4)	553 (42,4)	914 (47,4)	
¿Considera que hay muchos casos de COVID-19 en su comunidad?					
	Nada o casi nada	1788 (55,3)	703 (53,9)	1085 (56,3)	0,192
	Mucho	1443 (44,7)	600 (46,1)	843 (43,7)	
¿Considera que lleva a cabo adecuadamente las medidas de prevención frente a la COVID-19?					
	Nada o casi nada	988(30,6)	373 (28,6)	615 (31,9)	0,048
	Mucho	2243 (69,4)	930 (71,4)	1313 (68,1)	
Opinión sobre el uso de plantas medicinales para prevenir la COVID-19					
	Es efectivo	1611 (49,9)	256 (19,7)	1355 (70,3)	<0,001
	No es efectivo	822 (25,4)	480 (36,8)	342 (17,7)	
	No está informado	798 (24,7)	567 (43,5)	231 (12,0)	
Uso de medicamentos para prevenir la COVID-19					
	No	2517 (77,9)	1208 (92,7)	1309 (67,9)	<0,001
	Sí	714 (22,1)	95 (7,3)	619 (32,1)	
Uso de dióxido de cloro para prevenir la COVID-19					
	No	2972 (92,0)	1253 (96,2)	1719 (89,2)	<0,001
	Sí	259 (8,0)	50 (3,8)	209 (10,8)	

*Calculado con prueba chi^2^; ** Las categorías no son excluyentes.

En cuanto a la percepción sobre la COVID-19, el 76,8% de los encuestados consideró que era muy peligrosa y mortal, el 74,9% pensó que tenía bajo o nulo riesgo de enfermarse por COVID-19, el 45,4% consideró que sus familiares estuvieron expuestos a un mayor riesgo de infectarse, el 44,7% afirmó que en su comunidad hubo muchos casos de COVID-19 y el 49,9% consideró que el consumo de plantas medicinales fue efectivo para prevenir la transmisión de COVID-19 (Tabla 1).

Se informó que el 59,7% consumió al menos una planta medicinal para prevenir la COVID-19, la más consumida fue el eucalipto (77,7%), kion (66,7%) y matico (44,6%), la descripción del total de plantas medicinales consumidas se presenta en el material suplementario 2. La mayor proporción de los consumidores fueron mujeres (65,8%, p<0,001), jóvenes (18-29 años) (83,2%, p=0,008), de educación superior (78,4%, p<0,001), de la región sierra (47,7%, p<0,001), con residencia urbana (82,3%, p<0,001), de clase media-alta (64,0%, p<0,001), que no contaba con un profesional de la salud dentro de la unidad familiar (43,8%, p<0,001), adquirió información sobre COVID-19 a través de redes sociales (38,1%, p<0,001), no tenía un familiar con diagnóstico (69,3%, p<0,001) ni fallecido de COVID-19 (98,2%, p=0,003). Además, quienes consideraron fuertemente a la COVID-19 como una enfermedad peligrosa y mortal (78,6%, p=0,003), que consideraron nada o poco que tomar medicamentos, plantas u otras sustancias protegen contra enfermar de COVID-19 (72,1%, p<0,001), quienes consideraron nada o poco que tenían un mayor riesgo de contraer COVID-19 (73,0%, p=0,003), quienes consideraron nada o poco que su familia estaba expuesta a un mayor riesgo de contraer COVID-19 (52,6%, p=0,005), quienes consideraron que realizaron adecuadamente las medidas preventivas contra la COVID-19 (68,1%, p=0,048), quienes consideraron que el consumo de plantas medicinales para prevenir la COVID-19 fue efectivo (70,3%, p<0,001) y que no utilizaron medicamentos (67,9%, p<0,001) ni dióxido de cloro para prevenir la COVID-19 (89,2%, p<0,001) (Tabla 1).

En cuanto a los factores asociados en el análisis ajustado, encontramos que el consumo de plantas medicinales se asoció con residir en la sierra peruana (RPa 1,13, IC 95%: 1,07 - 1,19), haber tenido un familiar cercano diagnosticado con COVID-19 (RPa 1,07, IC 95%: 1,01 - 1,12), haber tenido un familiar fallecido por COVID-19 (RPa 1,26, IC 95%: 1,08 - 1,45), considerar que su familia tiene mayor riesgo de infección (RPa 1,06, IC 95% 1,01 - 1,12), haber usado medicación para prevenir la COVID-19 (RPa 1,33, IC 95%: 1,27 - 1,39) o haber usado dióxido de cloro para prevenir la COVID-19 (RPa 1,13, IC 95%: 1,06 - 1,21), tener información médica como fuente primaria de información sobre COVID-19 (RPa 0,81, IC 95%: 0,74 - 0,87), y pensar que las plantas medicinales son efectivas para prevenir la COVID-19 (RPa 1,17; IC 95% 1,11 - 1,22) así como no estar informado de su eficacia (RPa 0,40, IC 95%: 0,36 - 0,45) (Tabla 2).

**Tabla 2:** Factores asociados al consumo de plantas medicinales como prevención de COVID-19 en una población peruana, 2020 (N=3231).

Variables		RP crudo	IC 95%	RP ajustado	IC 95%
Sexo					
	Femenino	1,00		1,00	
	Masculino	0,87	0,82 - 0,93	0,96	0,91 - 1,01
Edad					
	Joven (18 a 29 años)	1,00		1,00	
	Adultos y adultos mayores (≥30 años)	1,11	1,03 - 1,19	1,07	0,98 - 1,17
Estado civil					
	Soltero	1,00		1,00	
	Casado o conviviente	1,08	0,99 - 1,17	0,96	0,88 - 1,05
Grado de educación					
	Escuela secundaria o inferior	1,00		1,00	
	Superior	1,14	1,06 - 1,23	1,06	0,99 - 1,12
Región					
	Costa	1,00		1,00	
	Sierra	1,25	1,18 - 1,33	1,13	1,07 - 1,19
	Selva	1,07	0,96 - 1,19	0,91	0,83 - 1,00
Residencia					
	Urbano	1,00		1,00	
	Rural	1,21	1,13 - 1,29	1,00	0,94 - 1,06
Situación laboral					
	Desempleado	1,00		1,00	
	Empleado	1,01	0,94 - 1,08	0,93	0,87 - 1,00
Estatus social					
	Bajo	1,00		1,00	
	Medio-alto	1,24	1,16 - 1,32	1,03	0,98 - 1,09
Profesional sanitario en familia					
	No	1,00		1,00	
	Estudiante de ciencias de la salud	0,92	0,85 - 0,99	0,98	0,92 - 1,05
	Sí	0,78	0,73 - 0,83	0,94	0,89 - 1,00
Fuente de información sobre COVID-19					
	Redes sociales	1,00		1,00	
	Medios de prensa	0,97	0,91 - 1,03	0,98	0,93 - 1,03
	Información médica	0,63	0,58 - 0,69	0,81	0,74 - 0,87
	Amigos y familiares	0,99	0,86 - 1,14	0,94	0,83 - 1,06
Comorbilidades para COVID-19					
	No	1,00		1,00	
	Sí	1,01	0,93 - 1,09	0,97	0,90 - 1,04
Familiares con comorbilidades para COVID-19					
	No	1,00		1,00	
	Sí	1,00	0,94 - 1,05	1,01	0,96 - 1,06
Familiares diagnosticados con COVID-19					
	No	1,00		1,00	
	Sí	1,27	1,20 - 1,34	1,07	1,01 - 1,12
Familiar fallecido por COVID-19					
	No	1,00		1,00	
	Sí	1,37	1,18 - 1,59	1,26	1,08 - 1,45
¿Considera la COVID-19 una enfermedad peligrosa y mortal?					
	Nada o casi nada	1,00		1,00	
	Mucho	1,11	1,04 - 1,20	1,05	0,98 - 1,12
¿Considera que utilizar medicamentos, plantas u otras sustancias te protege de la COVID-19?					
	Nada o casi nada	1,00		1,00	
	Mucho	1,62	1,55 - 1,70	1,17	1,11 - 1,22
¿Considera que tiene mayor riesgo de contagiarse de COVID-19?					
	Nada o casi nada	1,00		1,00	
	Mucho	1,10	1,03 - 1,17	1,05	0,99-1,12
¿Considera que su familia está expuesta a un mayor riesgo de contraer COVID-19?					
	Nada o casi nada	1,00		1,00	
	Mucho	1,08	1,02 - 1,15	1,06	1,01 - 1,12
¿Consideras que hay muchos casos de COVID-19 en tu comunidad?					
	Nada o casi nada	1,00		1,00	
	Mucho	0,96	0,91 - 1,02	0,96	0,91 - 1,01
¿Considera que lleva a cabo adecuadamente las medidas de prevención frente a la COVID-19?					
	Nada o casi nada	1,00		1,00	
	Mucho	0,94	0,89 - 1,00	0,96	0,91 - 1,01
Opinión sobre el uso de plantas medicinales para prevenir la COVID-19					
	Es efectivo	1,00		1,00	
	No es efectivo	0,49	0,45 - 0,54	0,60	0,55 - 0,66
	No está informado	0,34	0,31 - 0,38	0,40	0,36 - 0,45
Uso de medicamentos para prevenir la COVID-19					
	No	1,00		1,00	
	Sí	1,67	1,59 - 1,75	1,33	1,27 - 1,39
Uso de dióxido de cloro para prevenir la COVID-19					
	No	1,00		1,00	
	Sí	1,40	1,30 - 1,49	1,13	1,06 - 1,21

RP: Razón de prevalencia; IC 95%: intervalo de confianza al 95%.

## DISCUSIÓN

La medicina herbaria se ha propuesto como una alternativa en el control y prevención de enfermedades infecciosas virales como el SARS-CoV2 y actualmente se ha propuesto como una alternativa para el manejo de la COVID-19 ^17^. Sin embargo, la información que aportan los estudios que valoran su eficacia es insuficiente o de baja calidad, siendo necesarios más estudios que recomienden su uso como prevención ^18^. En Perú, un país con gran riqueza y diversidad histórica, cultural y natural, el consumo de plantas medicinales ha sido la principal práctica cultural vinculada al cuidado de la salud ^19^. 

Nuestro estudio encontró que más de la mitad de la población reportó haber consumido alguna planta medicinal para prevenir la COVID-19, similar a estudios previos realizados en población peruana antes de la pandemia ^18^^,^^20^. Este resultado fue inferior al 80% reportado en un estudio realizado en Cusco, Perú ^15^ y más del 14,1% y 7,2% en usuarios de Facebook de Perú y América Latina, respectivamente ^21^. Estas variaciones pueden dilucidarse considerando el contexto geográfico; el primer estudio se realizó en Cusco, una región andina caracterizada por un mayor consumo de plantas medicinales. La divergencia en el segundo estudio podría deberse a diferencias en la población y en los procedimientos de recopilación de datos.

Asimismo, la proporción es similar a la reportada en Banglaseh ^14^, pero diferente de la encontrada en Arabia Saudita ^22^, donde el 57,6% y el 22,1% de las poblaciones de los estudios consumieron plantas medicinales para la prevención de la COVID-19, respectivamente. Se debe tener en cuenta que la creencia en las propiedades preventivas de las plantas medicinales puede estar relacionada con factores culturales y sociales como el nivel de educación, la ocupación e ingresos económicos de los participantes ^18^^,^^23^. Estos factores sumados a una mayor accesibilidad y menor costo respecto a los productos farmacéuticos ^24^, constituirían la base para un mayor consumo de plantas medicinales por parte de la población que las considera efectivas.

En nuestro estudio encontramos que vivir en la sierra se asociaba con un mayor consumo de plantas medicinales, donde su uso y conocimiento para fines terapéuticos es una práctica ancestral ^24^, transmitida de generación en generación ^25^, constituyendo un importante componente cultural. Estudios etnobotánicos informaron que el conocimiento y uso de las plantas medicinales aumenta directamente con la proximidad entre las personas y las fuentes disponibles ^26^^,^^27^. Por lo tanto, teniendo en cuenta que un gran porcentaje de las plantas medicinales utilizadas en el Perú se encuentran en esta región ^28^, se espera que la población que vive en la sierra muestre un mayor consumo de plantas para uso medicinal.

Por otro lado, encontramos que estar empleado se asoció con un menor consumo de plantas medicinales, similar a un estudio en México donde se encontró que estar desempleado se asoció con un mayor consumo de plantas medicinales ^29^. Aunque las plantas medicinales tienen un bajo costo económico ^30^, el acceso al trabajo y, en consecuencia, mayores recursos pueden hacer que las personas prefieran métodos de prevención más costosos, pero ampliamente publicitados. Asimismo, el pánico por enfermar podría ser una variable mediadora en esta relación, ya que las personas con empleo tienen mayor estabilidad económica y recursos sanitarios y, por tanto, pueden estar menos ansiosas por su salud ^31^. Lo que, finalmente, puede llevarlos a un menor consumo de plantas medicinales.

Los medios de comunicación tienen un rol crucial en la sensibilización pública sobre la COVID-19, pero también han generado pánico y transmitido información errónea, lo que ha provocado que la gente recurra al uso de medidas con escaso o nulo reconocimiento científico ^32^^,^^33^, En el presente estudio se encontró que tener información médica como principal fuente de información sobre la COVID-19 se asoció con un menor consumo de plantas medicinales para su prevención. En el Perú, aunque se ha reportado que más de las tres cuartas partes de los pacientes han utilizado plantas medicinales, la proporción de médicos que aconsejan su uso es mínima ^7^. Probablemente esto se deba a que la formación de los médicos está principalmente orientada al uso de la medicina occidental, lo que generaría una mínima inclusión de plantas medicinales entre sus recomendaciones. Por lo mismo, se esperaba que contar con profesionales de la salud en la unidad familiar también se asociaría con un menor consumo de plantas medicinales.

Por otro lado, considerar que la familia está expuesta a un mayor riesgo de contagio, tener un familiar diagnosticado con COVID-19 y que el familiar haya fallecido, se asociaron con un mayor consumo de plantas medicinales, resultado similar a un estudio realizado en Cusco ^15^. Estas situaciones aumentan el temor y preocupación de la población ante la enfermedad, lo que podría llevar a la aplicación de más de una estrategia de prevención como el uso de plantas medicinales, independientemente del conocimiento de su eficacia ^34^, como se informó en un estudio previo ^21^.

También se observó que las personas que consumieron medicamentos o dióxido de cloro para prevenir la COVID-19 consumieron más plantas medicinales que quienes no lo hicieron. Este resultado es similar al reportado en otros estudios donde es común el uso concomitante de plantas medicinales y fármacos ^35^^,^^36^. Sin embargo, esta práctica de consumo puede ser riesgosa debido a las posibles interacciones que puedan existir ^37^. Aunque no hemos encontrado estudios clínicos al respecto, un estudio in vitro reportó una mayor prevalencia de hepatotoxicidad debido a la interacción del paracetamol, fármaco comúnmente utilizado durante la pandemia, y compuestos herbales que contienen furanocumarinas ^38^.

Hay que tener en cuenta que el consumo de plantas medicinales como prevención de la COVID-19 aún carece de estudios de calidad que aseguren su eficacia, seguridad y rentabilidad ^15^^,^^39^. Por ello, recomendamos promover capacitaciones, charlas, etc. que contengan información sobre prácticas preventivas validadas y científicamente respaldadas para la población, así como de los riesgos de su consumo, utilizando un enfoque individualizado según las características de los consumidores predominantes. Se deberían realizar estudios futuros que corroboren los resultados del presente estudio, así como estudios cualitativos para comprender mejor este fenómeno.

El presente estudio tuvo ciertas limitaciones. Se empleó un método de muestreo no probabilístico para reclutar participantes, lo que no permite la extrapolación de los resultados. Dado que la recolección de datos se realizó a través de un cuestionario virtual distribuido a través de redes sociales, este podría haber sido respondido, en su mayor parte, por una población con acceso y conocimiento de estos medios, por lo que nuestros resultados resaltarían la perspectiva de un único estrato sociocultural. Sin embargo, nuestros resultados muestran una realidad en el contexto de la pandemia de COVID-19 poco abordado en el Perú, siendo un país con una riqueza histórica y cultural en plantas medicinales.

En conclusión, más del 60% de los participantes refirieron consumir alguna planta medicinal para buscar prevenir la COVID-19. Residir en la sierra, tener un familiar diagnosticado o fallecido por COVID-19, considerar que la familia tiene mayor riesgo de contagio y haber utilizado medicamentos y dióxido de cloro para la prevención se asociaron con un mayor consumo de plantas medicinales. Es importante conocer y comprender el consumo de plantas medicinales ya que, como cualquier otra sustancia sin evidencia médica completa, podrían provocar efectos adversos o una falsa sensación de seguridad.
